# Application of high-resolution mass spectrometry profiling towards the diagnosis and acute management of maple syrup urine disease

**DOI:** 10.1016/j.ymgmr.2025.101250

**Published:** 2025-09-12

**Authors:** Rafael Garrett, Sara Pickett, Melinda J. Peters, Khadija Belhassan, Adam S. Ptolemy, Roy W.A. Peake

**Affiliations:** a Department of Laboratory Medicine, Boston Children's Hospital, Harvard Medical School, Boston, MA, USA; b Division of Genetics and Genomics, Boston Children's Hospital, Harvard Medical School, Boston, MA, USA; c Federal University of Rio de Janeiro, Institute of Chemistry, Metabolomics Laboratory, Rio de Janeiro, Brazil

**Keywords:** MSUD, Inborn error of metabolism, Metabolomics, Mass spectrometry

## Abstract

The current approach for investigating patients with suspected inborn errors of metabolism (IEMs) involves traditional targeted biochemical assays such as amino/organic acid analyses. Although highly effective for confirmatory testing, they are less effective in identifying disorders not included in newborn screening (NBS) panels, and for patients with non-classical clinical presentations. Targeted assays analyze a narrow range of metabolites and are conducted across different analytical platforms, often requiring more than one specimen type. In contrast, comprehensive metabolic profiling using liquid chromatography-high-resolution mass spectrometry (LC-HRMS) provides significantly more information from a single specimen, eliminating the need for multiple and time-consuming analyses across different platforms. We describe the use of LC-HRMS metabolic profiling in two patients with decompensated maple syrup urine disease (MSUD). In the first patient, a previously healthy 3-month-old infant presenting with altered mental status, apnea, and seizures, LC-HRMS analysis of plasma before treatment showed increased levels of branched-chain amino acids and their related 2-keto and hydroxy acids. The diagnosis of MSUD was confirmed by targeted amino acid analysis. Additionally, the treatment course, which included dialysis and nutritional management, was monitored using LC-HRMS. This approach was successfully applied to a second patient, a 1-week-old infant with classical MSUD identified through NBS. In conclusion, comprehensive metabolic profiling by LC-HRMS is a valuable investigative tool for patients with both classic and non-specific neurometabolic clinical phenotypes, providing additional insights into metabolite perturbations during acute management.

## 1 Introduction

Inborn errors of metabolism (IEMs) are a diverse group of over 1000 genetic disorders of wide phenotypic variation [1]. A large number of IEMs require early recognition, diagnosis, and treatment in order to prevent irreversible clinical sequelae. As such, newborn screening (NBS) programs in the U.S. have expanded to incorporate approximately 50 IEMs in the list of recommended disorders [2]. Despite this, many treatable IEMs are not identifiable through NBS and must be recognized through clinical evaluation by metabolic specialists at tertiary referral centers. The work-up of such patients involves so-called “traditional” biochemical screening of plasma, urine, and cerebrospinal fluid (CSF) specimens using the standard trio of targeted screening tests, namely amino acid, carnitine-acylcarnitine, and organic acid analyses. The limitations of this approach are apparent, with one recent study reporting a diagnostic yield of only 1.3 % in patients with a strong suspicion of a genetic/neurometabolic disorder or syndrome [3]. Therefore, it may be inferred that a significant number of individuals with undiagnosed IEMs are either being missed or, at best, having their diagnosis delayed. Of course, additional targeted biochemical assays beyond the aforementioned trio of tests are available; however, these tests are performed by a decreasingly small number of specialized laboratories due to their complexity and are often not considered, even by genetic-metabolic specialists. Given the phenotypic heterogeneity of IEMs, recognizing the most appropriate targeted biochemical investigation is not straightforward, even for specialists. This often results in patients being referred to multiple clinical services and a diagnostic odyssey that can last for several years [4]. This is especially true for patients with non-classical IEMs with milder, intermittent, or attenuated disease.

The use of liquid chromatography-high-resolution mass spectrometry (LC-HRMS)-based metabolomics in routine biochemical laboratories has been of interest for over a decade [[5], [6], [7], [8]]. LC-HRMS enables a more comprehensive assessment than is possible with traditional biochemical screening methods, with potentially hundreds of metabolite perturbations detected in a single specimen. Not only is this approach more efficient by avoiding the need to perform several sequential targeted assays, but it also allows retrospective evaluation for research purposes, since the full scan data can be revisited post-acquisition. Thus, LC-HRMS metabolomics has emerged as a powerful tool for disease screening and biomarker discovery [9]. The addition of comprehensive IEM screening by LC-HRMS would provide routine biochemical genetics laboratories with broader capabilities than are currently available in most centers, potentially increasing the diagnostic yield for rare diseases [10].

**Fig. 1:**
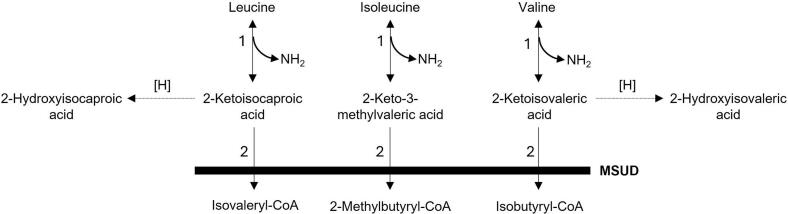
Branched-chain amino acid catabolism. The initial two reactions are catabolized by the following enzymes: 1. Branched-chain aminotransferases (reversible); 2. The common mitochondrial branched-chain keto-acid dehydrogenase (BCKAD) complex. Deficiency of the BCKAD complex results in MSUD and the accumulation of 2-ketoacids (2-ketoisocaporic, 2-keto-3-methylvaleric, and 2-ketoisocaleric acids) and their respective progenitor branched-chain amino acids (leucine, isoleucine, and valine). 2-Keto acids may undergo further reduction to 2-hydroxyacids, such as 2-hydroxyisovaleric acid and 2-hydroxyisocaproic acid (leucic acid).

Maple syrup urine disease (MSUD, OMIM # 248600) is a life-threatening metabolic disorder caused by biallelic pathogenic variants in the *BCKDHA*, *BCKDHB, DBT*, and *DLD* genes, which encode the E1α, E1β, E2 and E3 subunits (respectively) of the branched-chain amino acid (BCKD) enzyme complex [11,12]. In MSUD, accumulation and exposure duration of neurotoxic leucine and the branched-chain ketoacids (BCKAs) (Fig. 1) directly correlate with brain damage [13]. As such, early detection (ideally in the pre-symptomatic stage) is imperative to avoid the toxicity of hyperleucinosis, and aggressive lowering of leucine levels in the body fluids is necessary. Hemodialysis (HD) is an established emergency treatment modality in MSUD that enables rapid clearance of leucine and its toxic metabolites [14].

Most patients with classical MSUD presentations are detected by NBS and diagnosed through standard confirmatory biochemical testing in referral centers [15]. However, non-classical variant MSUD sub-types (including intermittent and intermediate forms) are more challenging to identify since they can be missed by NBS and typically have delayed onset with variable symptoms that may not immediately prompt a request for amino acid analysis [16]. In such cases, comprehensive metabolite screening may be beneficial. We investigated two critical patients presenting with MSUD: i) a 3-month-old infant with variant MSUD who presented with new onset seizures, and ii) a 3-day-old baby with classical MSUD detected by NBS. Comprehensive metabolic profiling of plasma samples, from before and throughout the treatment course, was performed by untargeted LC-HRMS in parallel to traditional targeted biochemical screening using conventional plasma amino acid analysis for diagnosis and management.

## 2 Materials and methods

### 2.1 Materials

Methanol and acetonitrile (Optima™ LC/MS Grade) and methyl tert-butyl ether (HPLC Grade) were obtained from Fisher Scientific (Waltham, MA). Ammonium acetate (LiChropur™, eluent additive for LC-MS) was from Sigma-Aldrich (St Louis, MO). Ammonium hydroxide solution (eluent additive for LC-MS, ≥ 25 % in H_2_O) was from Honeywell Fluka (Charlotte, NC). High-purity water (18.2 MΩ∙cm) was obtained from a Millipore Milli-Q IQ Water Purification System. Stable isotope-labeled and non-labeled internal standards were from Cambridge Isotope Laboratories or Sigma-Aldrich.

### 2.2 Specimens

All plasma specimens used in this study were collected into lithium heparin tubes (Vacutainer®, PST™) (Becton Dickinson, Franklin Lakes, NJ) and submitted to our Core Laboratory for routine testing. Remnant plasma specimens from a non-classical MSUD patient (patient I) and a patient with classical MSUD presentation (patient II), along with 24 controls, which include 20 non-MSUD infants and 4 dietary-treated MSUD patients from age 0–1 years, were included in this study. This study was approved by the Boston Children's Hospital Institutional Review Board (IRB—P00041049).

### 2.3 Patient I

A previously healthy full-term 3-month-old male child presented following an episode of altered mental status and was initially diagnosed with febrile seizure. After 2 days of further shaking episodes, the child presented to the emergency department and underwent neurological evaluation and electroencephalogram monitoring. Plasma specimens were submitted for routine amino acid analysis and comprehensive screening analysis using LC-HRMS. Routine plasma amino acid analysis demonstrated marked increases in branch-chain amino acids (BCAA) leucine, isoleucine, valine, and allo-isoleucine. A genetic diagnosis of non-classical (intermittent) MSUD due to compound heterozygous variants in the *DBT* gene was ultimately confirmed (NM_001918.5(DBT):c.52-1G > A, p.(?)/ NM_001918.5(DBT):c.670G > T, p.(Glu224*). The infant was transferred to the neonatal intensive care unit (NICU) and underwent urgent hemodialysis (HD), followed by continuous renal replacement therapy (CRRT) and nutritional support. A total of 16 plasma specimens were collected prior to, during, and post-dialysis for LC-HRMS analysis.

### 2.4 Patient II

A 2-day-old term female with an uncomplicated pregnancy and birth history was flagged with an abnormal leucine newborn screen result. Based on a provisional diagnosis of MSUD, the infant was commenced on BCAA-free nutritional support. Plasma specimens collected for amino acid analysis revealed marked increases in BCAA, consistent with MSUD. A genetic diagnosis of classical MSUD due to compound heterozygous variants in the *BCKDHA* gene was ultimately confirmed (NM_000709.4:c.808 G > A, p.(Ala270Thr)/ NM_000709.4:c.929C > G, p. (Thr310Arg)). The child was initiated on HD followed by CRRT, and a total of 15 plasma specimens were collected throughout the course of treatment for LC-HRMS analysis.

### 2.5 Sample preparation

Samples were prepared according to a modified Matyash protocol [17]. 50 μL of plasma was combined with 150 μL of an ice-chilled methanol solution containing isotopically-labeled internal standards (IS) across the concentration range of 2 to 30 μg/mL (L-leucine-d_3_, l-lysine-d_4_, L-methionine-d_3_, *L*-phenylalanine-d_5_, cholic acid‑*d*_4_, alpha-ketoisovaleric acid-^13^C_5_, hippuric acid‑*d*_5_, creatinine-d_3_, succinic acid‑*d*_4_, and glutaric acid‑*d*_4_). Samples were vortex-mixed for 10 s, and 500 μL of ice-chilled methyl tert-butyl ether (MTBE) was added, followed by 5 min sonication in an ice-water bath. Next, 175 μL of ice-chilled ultrapure water was added, samples were vortex-mixed for 10 s and centrifuged at 12,000 x*g* for 15 min at 4 °C (Eppendorf Centrifuge 5430R). After standing for 1 min, 200 μL of the lower aqueous phase was transferred to another tube and dried in a centrifuge concentrator (Eppendorf Vacufuge Plus) at 45 °C, followed by reconstitution in 60 μL of an ice-chilled acetonitrile-water (80:20 *v*/v) solution containing a second set of IS across the concentration range of 1.0–5.0 μg/mL (4-*F*-phenylalanine, 5-Br-tryptophan, and 5-F-uridine). Ultrapure water was used for blank extraction, and an in-house plasma reference material based on the IBAT method was used for quality control and sample normalization [18]. 50 μL of each reconstituted sample was transferred into a Waters 12 × 32 mm Total Recovery Vial for liquid chromatography-high resolution mass spectrometry (LC-HRMS) analysis, and 5 uL of each sample extract was pooled to create an intra-assay quality control sample (QC).

### 2.6 Instrumental analysis

LC-HRMS was performed using an ACQUITY UPLC H-Class coupled to a Xevo G2-XS Q-Tof mass spectrometer (Waters Corp., Milford, MA) with an electrospray ionization (ESI) source operating in negative ion mode. Chromatographic separation was performed using an ACQUITY Premier BEH Amide column (2.1 mm × 100 mm; 1.7 μm) (Waters Corporation, Milford, MA) maintained at 40 °C and with a solvent flow rate of 400 μL/min. Mobile phases were (A) water and (B) 95:5 acetonitrile:water, both containing 10 mM ammonium acetate (pH 9 adjusted with ammonium hydroxide solution). The gradient elution program was as follows: 0–0.5 min B 99 %; 0.5–10.0 min B 99–40 %; 10–12.0 min B 40 %; 12.0–12.5 min B 40–99 %; and 12.5–17.0 min B 99 %. Sample temperature was maintained at 10 °C, and the injection volume was 5.0 μL. The mass spectrometer was operated in sensitivity analyzer mode with a capillary voltage of −1.5 kV, cone voltage of 25 V, source and desolvation gases temperatures of 120 and 500 °C, respectively, and cone and desolvation gases (nitrogen) flow rates of 20 and 800 L/h, respectively. Data acquisition was performed on MassLynx version 4.1 in centroid mode using the MS^E^ experiment in the *m/z* range of 50–1000, scan time of 0.2 s, low collision energy (CE) of 6 eV, and high CE ramp of 10–35 eV [18]. Routine amino acid analysis was carried out using an established quantitative amino acid method [19].

### 2.7 Data processing and statistical analysis

LC-HRMS raw files were imported into Skyline software v24 [20] and processed in a targeted fashion using an in-house library of selected IEM biomarkers, which contained experimental retention time, precursor, and fragment *m/z* values. Robust Standardized (RS) values were calculated for targeted metabolites according to Piskláková et al. [21] and were used to highlight abnormal metabolites in samples. Metabolite peak areas in MSUD samples prior to treatment were normalized to our plasma reference material (RM) [19], and their RS values were calculated based on computed reference values from a group of previously analyzed and normalized control samples (*n* = 146). Key metabolites related to MSUD with high RS values were monitored throughout patient treatment, and their area ratio (metabolite peak area to L-leucine-d_3_) was plotted against the treatment course in chronological order using GraphPad Prism v8.

In addition, untargeted data processing was performed using Progenesis QI v3.0 (Nonlinear Dynamics). For this data analysis, 16 plasma samples from patient I and 24 controls (20 non-MSUD infants and 4 dietary-treated MSUD patients from age 0–1 years) were used. Peak picking parameters were default automatic sensitivity and a minimum peak width of 0.08 min. Fold change ≥2 and ANOVA *P*-value ≤0.05 criteria were used to select significant discriminant features for compound annotation, which was performed using the Progenesis MetaScope search engine with the HMDB 5.0 and Lipid Maps databases considering an *m/z* error below 10 ppm. The aligned data table with samples in rows and features (pair of *m/z*-t_R_) in columns was exported as a .csv file, processed in QCMXP v1.4 for pooled QC-based intra-batch correction and data cleaning [22], and analyzed in Orange Data Mining v3 [23] for multivariate statistical analysis.

## 3 Results and discussion

**Fig. 2:**
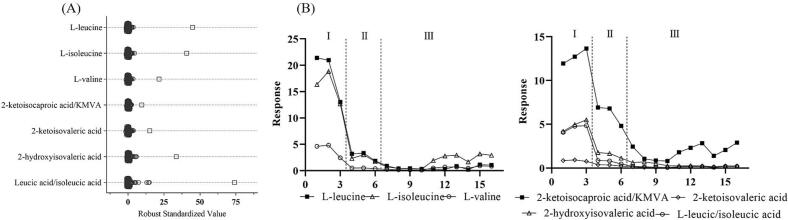
(A) Robust standardized value plot for BCAAs and 2-keto/hydroxy acids analogues detected in patient I plasma prior to treatment (square) compared to controls (circles). (B) Response levels of BCAAs and 2-keto/hydroxy acids analogues (y-axis) detected in patient I plasma samples in chronological order (x-axis). Phase I: pre-dialysis; phase II: dialysis; phase III: post-dialysis/renal replacement therapy/nutritional management. 2-ketoisocaproic and KMVA (2-keto-3-methylaleric acid), as well as leucic acid (2-hydroxyisocaproic acid) and isoleucic acid (2-hydroxy-3-methylpentanoic acid) are isomers that coelute in the untargeted LC-HRMS method.

LC-HRMS metabolic screening was conducted on pre-treatment plasma in parallel with routine amino acid analysis to investigate patient I. An untargeted acquisition method was employed, combining full scans at low and high fragmentation energies (Waters MS^E^), followed by data processing in Skyline using an in-house library of IEM biomarkers associated with various diseases. This approach enabled us to screen for a broad range of metabolites across the major compound classes, including (but not limited to) amino acids, organic acids, acylcarnitines, bile acids, and sugars in a single injection of patient plasma. Fig. 2A shows a dot plot of Robust Standardized (RS) values calculated for abnormal metabolites found in plasma from patient I based on the median and interquartile range of 146 controls that were previously injected and normalized through the IBAT method [18]. RS was chosen as a scaling method due to its robustness when dealing with skewed data, being less sensitive to outliers compared to z-score determinations. As illustrated in Fig. 2A, BCAAs and their toxic products 2-keto/hydroxy acid analogues exhibited increased RS values compared to controls. This profile is consistent with a diagnosis of MSUD, in which the branched-chain α-ketoacid dehydrogenase (BCKD) complex responsible for the catabolism of BCAAs is deficient, causing the accumulation of leucine, isoleucine, and valine in blood, as well as their metabolic products [24]. The diagnosis was confirmed by routine plasma amino acid analysis, which showed a markedly increased leucine concentration at 2295 μmol/L (reference interval: 32–153). Subsequent molecular analysis revealed a splice site c.52-1G > A, p.(?) variant and a nonsense c.670G > T, p.(Glu224*) variant in the *DBT* gene. The clinical presentation would suggest a non-classical (intermittent) MSUD phenotype. Previously published functional studies on these variants demonstrated premature chain termination immediately downstream of the E1/E3 binding domain, resulting in a mutant E2 and reduced BCKAD activity [25,26]. In lieu of enzymology data, the quantitative and/or qualitative effect on the function of the BCKAD complex is unclear. As such, there is interest in metabolic profiling as a tool to assess functional effects of variants in such cases where molecular data cannot adequately predict the outcome of enzyme-subunit interactions, nor explain the phenotype. Subsequent molecular analysis revealed compound heterozygous variants in the *DBT* gene, consistent with a non-classical (intermittent) form of MSUD [27]. Notably, comprehensive LC-HRMS metabolite screening is novel in that it is able to assess BCKAs and hydroxy-acid derivatives together with their precursor amino acids. Although clinical methods for blood organic acids have been described, these are targeted assays for a single compound class, and are unable to assess amino acids, for example. Additionally, the dominance of established urine organic acid methods using gas chromatography mass spectrometry has greatly limited the introduction of blood organic acid methods for IEM screening. Identification of these latter compounds is possible by qualitative urine organic acid analysis. Assessment of this type of data on a single plasma specimen is advantageous, given the challenges associated with collecting urine specimens from neonates and infants. Additionally, since BCKAs and their primary metabolic by-products (2-hydroxy acids) represent a major source of the neurotoxic effects in MSUD patients, their assessment in blood (particularly during acute episodes of the disease) together with amino acids provides additional scope and information on the recovery of MSUD patients following metabolic decompensation and their response to treatment. This is particularly true for blood levels of BCKAs, since they provide a more suitable surrogate of levels within the central nervous system than urine, for example. Additionally, other dietary confounders (such as thiamine) may be relevant factors in driving non-classical presentations, as in this patient.

In accordance with local guidelines on the treatment of hyperleucinosis in MSUD, patient I underwent urgent hemodialysis (HD) followed by continuous renal replacement therapy (CRRT) and titration of nutritional support. Plasma specimens were collected prior to (I), during (II), and post-dialysis (III) for LC-HRMS analysis. Fig. 2B shows a line plot of metabolite response (ratio of metabolite peak area to leucine-d_3_) *versus* treatment in chronological order. In phase I (pre-HD), BCAAs concentrations were at maximum blood concentrations. During this phase, the patient was receiving special metabolic formula (Ketonex-1) with the offending BCAAs omitted in an effort to reduce blood leucine levels, which resulted in a modest decrease in BCAA; notably, this reduction was not observed with the 2-keto/hydroxy acid analogues, which continued to rise prior to the initiation of HD. Phase II, which represented the metabolite response to HD and CRRT, effectively lowered toxic levels of all metabolites. However, although the blood concentration of neurotoxic leucine was rapidly decreased, the elimination of its highly toxic product, 2-ketoisocaproic acid (2-KIC), appeared partially blunted. Phase III represents the post-HD portion of treatment, primarily based on protein titration in order to stabilize blood BCAA levels whilst providing adequate nutrition. During this phase, both BCAA and BCKAs fluctuated together at lower levels compared to the pre-treatment samples, indicating successful nutritional and medical management. These results are in agreement with previous reports regarding dialysis intervention in MSUD patients [14,28].

**Fig. 3:**
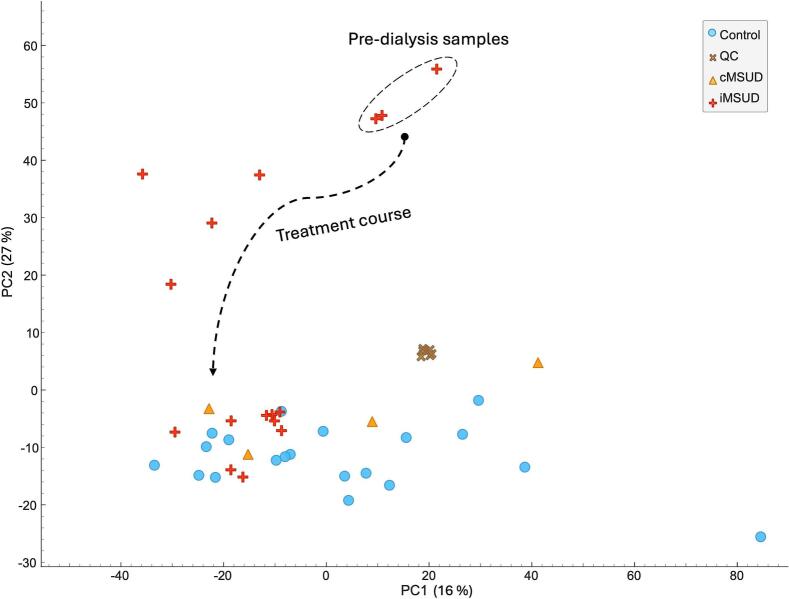
Principal component analysis scatter plot of patient I samples and controls from the untargeted LC-HRMS data processing. Circles: control samples from non-MSUD infants; plus: patient I samples; triangle: control samples from treated MSUD patients; cross: pooled quality control samples.

For a more global assessment of the acute metabolic state in our MSUD patient, specimens were processed in an untargeted fashion and compared with plasma from four stable classical MSUD patients on established treatment regimens, and 20 non-MSUD control plasma specimens collected from age-matched infants. A principal component analysis (PCA) scatter plot revealed considerable heterogeneity in the metabolome of patient I specimens, indicating a continuous metabolic change throughout the treatment course. (Fig. 3). The PCA was constructed using a data matrix of 3652 features (detected signals corresponding to a *m/z* and retention time pair). Therefore, the distribution profile of patient I samples, which reflects its global metabolic state, goes far beyond the targeted BCAAs and their metabolic products described for the targeted data processing. Specimens were dispersed across the PCA plot and clustered into three groups according to the phase of treatment, similar to that described in Fig. 2. Specimens collected in phase I (acute decompensation with formula management, pre-HD) were clustered and located close to specimens from phase II (dialysis phase), although they were separated by PC1. These two groups are distant and separated by PC2 from the specimens collected in phase III (post-HD), which represented the successful clearance of the offending metabolites and stabilization through the re-introduction of protein. During this phase, patient I specimens clustered with the control group, as desired, confirming the treatment efficacy. Additionally, quality control (QC) samples (which consisted of small aliquots from all samples after extraction and were injected repeatedly throughout the batch at a frequency of every 10 samples) were tightly clustered, demonstrating the reproducibility of the LC-HRMS system [29]. Metabolites beyond the targeted BCAAs and associated metabolites that could be annotated as responsible for discriminating the treatment phases, considering fold change ≥2 and ANOVA *P*-value ≤0.05, were glutamine, *N*-acetyl-leucine/isoleucine, γ-glutamyl-leucine/isoleucine, phenyl-lactic acid, and 4-hydroxyphenyl-lactic acid. Increased levels of the first two metabolites have been previously described in MSUD patients [30,31].

**Fig. 4:**
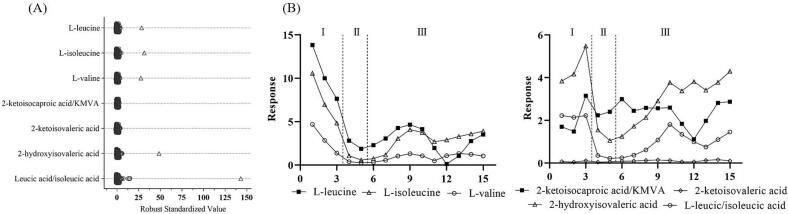
(A) Robust standardized value plot for BCAAs and 2-keto/hydroxy acids analogues detected in the plasma sample of patient II prior to dialysis and on BCAA-free formula (square) compared to controls (circles). (B) Response levels of BCAAs and 2-keto/hydroxy acids analogues (y-axis) detected in patient II plasma samples in chronological order (x-axis). Phase I: pre-dialysis; phase II: dialysis; phase III: nutrition support. 2-ketoisocaproic and KMVA (2-keto-3-methylvaleric acid), as well as leucic acid (2-hydroxyisocaproic acid) and isoleucic acid (2-hydroxy-3-methylpentanoic acid) are isomers that coelute in the untargeted LC-HRMS method.

The second patient (patient II), who presented with hyperleucinosis after identification through newborn screening (NBS), was confirmed as having MSUD by targeted plasma amino acid analysis. Notably (and in contrast to patient I), patient II was provisionally diagnosed as having MSUD immediately after the abnormal NBS result and was already receiving BCAA-free nutritional support (Ketonex-1) on arrival at our institution. Plasma specimens collected at baseline were analyzed by routine amino acid analysis and confirmed a markedly increased leucine level at 1581 μmol/L (reference interval: 32–153), providing confirmation of an MSUD diagnosis. In a similar approach to that employed for patient I, LC-HRMS followed by targeted data processing was performed throughout the course of treatment and revealed increased RS values for the BCAAs and their analogues 2-hydroxy acids compared to control samples (Fig. 4A). Notably, BCKA RS values were within the control range, likely due to the ongoing nutritional support already implemented based on the provisional diagnosis of MSUD. This early treatment also resulted in a significant reduction in blood BCAA levels prior to HD (phase I, Fig. 4B), which subsequently reduced the levels of all metabolites (phase II, Fig. 4B). A similar trend was also observed in patient I (phase II, Fig. 2B). Following HD (phase III), patient II was managed with a combination of parenteral nutrition, enteral diet, and protein titration resulting in clinical and nutritional stabilization.

## 4 Discussion

For decades, the diagnosis of IEMs has relied on traditional targeted biochemical analyses using various chromatography-mass spectrometry-based analytics. The major limitation of this approach is requirement of *a priori* hypothesis, which typically takes the form of either, (i) an abnormal NBS result, (ii) a characteristic clinical phenotype for a specific disorder or group of disorders, and (iii) the presence of disease-causing variants in a causative gene requiring functional validation. Such situations require targeted biochemical testing on several specimen types across multiple analytical platforms. This is problematic for several reasons; firstly, the vast majority of IEMs are not screened for at birth and must be identified clinically. This is particularly challenging with atypical or non-classical forms of IEMs, which are easily missed by clinicians either by failure to recognize the clinical phenotype, or to select the most appropriate test. Secondly, obtaining multiple specimen types (and urine in particular) from neonates and infants is not always possible, particularly in acute presentations where metabolic disease work-up is important in determining patient management. Lastly, it is no longer feasible for biochemical genetics laboratories in academic medical centers to maintain a complete array of targeted assays available on-site. Our metabolomics approach would not only minimize the requirement for multiple specimen types but broaden the number of compounds and disorders screened up-front, which not only relieves the burden on providers, but has the potential to shorten the diagnostic odyssey in patients with rare, undiagnosed IEMs.

In this study, we demonstrated the practical application of our LC-HRMS metabolic profiling workflow in two very different clinical presentations of MSUD. In both cases, our method revealed increased levels in the pathognomonic BCAAs (leucine, isoleucine, and valine) in a single plasma specimen, correctly identifying MSUD. This was particularly relevant for our patient with atypical MSUD, a diagnosis associated with high morbidity and mortality that was not initially suspected based on his presenting symptoms. If comprehensive metabolic screening is liberally deployed in the work-up of such patients with broad clinical phenotypes, we predict that this will increase diagnostic yield and decrease the time to diagnosis for rare diseases. Also, despite our LC-HRMS method offering only “semi-quantitative” data, it correctly identified our second patient with MSUD based on increased BCAA levels, and the biochemical response to treatment in both patients mirrored the established quantitative method. Our LC-HRMS method also identified the neurotoxic 2-keto and 2-hydroxy-acid metabolites of BCAAs, which were also monitored in plasma using LC-HRMS during dialysis treatment in both patients. These metabolites are not measured using standard routine biochemical assays, and their distinct clearance profiles compared to their BCAA precursors may provide additional insights into the toxicity of MSUD and the metabolic response to hemodialysis. This was particularly informative in this case since the clearance of 2-KIC appeared slower than its precursor leucine. This phenomenon may be simply due to disruption of the transamination equilibrium by hemodialysis, or perhaps this may provide new evidence that keto-acids in MSUD are disproportionately increased relative to their BCAA precursors. And finally, since LC-HRMS analysis was performed on a single plasma specimen, thereby avoiding the need for separate targeted biochemical assays on both plasma and urine specimens. Although it is not likely that LC-HRMS methods are ready to replace traditional amino acids for monitoring the acute response to treatment in MSUD patients, for example, this study showcased its correlation with the latter, and the additional information that may be gained from a single analysis.

**Fig. 5:**
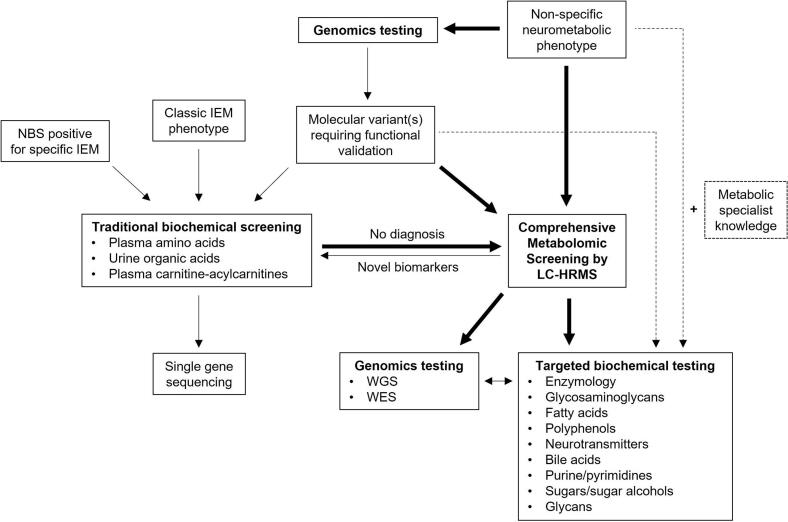
Proposed interplay of biochemical and genomics testing for IEMs. The role of traditional biochemical assay screening will continue to evolve towards confirmatory tools for NBS positive cases and for those with a clear phenotype consistent with a specific IEM. Comprehensive metabolic screening using LC-HRMS will evolve into the major investigative pathway (represented by bold arrows) to be used in the diagnostic work-up of non-specific neurometabolic phenotypes and functional validation of molecular variants of unclear clinical significance. This paradigm shift will relieve the burden on metabolic specialists in selecting the correct form of targeted biochemical testing for a given non-specific presentation or equivocal molecular variant identified by genomics testing (represented by the dotted arrows).

Comprehensive metabolomics profiling using LC-HRMS has emerged as a powerful tool for the investigation of rare diseases, including IEMs. This approach is not intended to replace the existing traditional targeted biochemical assays, but rather a complementary investigation with application for disorders not covered in State newborn screening programs, for broad biochemical assessment of patients presenting with non-specific neurometabolic phenotypes, and for atypical or non-classical presentations of IEMs, all of which may escape detection and result in diagnostic odyssey (Fig. 5). It may also have an important role in the functional assessment of genetic variants of uncertain clinical significance in pathogenic genes associated with IEMs. Our data demonstrate that LC-HRMS may be used as a screening investigation for IEMs, deployed in the aforementioned clinical scenarios.

## 5 Conclusion

This study demonstrates the effectiveness of comprehensive plasma metabolic profiling using LC-HRMS as a powerful diagnostic and investigative tool for inborn errors of metabolism (IEMs). We have also demonstrated its utility in patients presenting with atypical forms of potentially fatal diseases that are not clinically recognizable, such as intermittent MSUD. The LC-HRMS approach successfully identified key metabolite perturbations in the BCAA pathway in two critical MSUD patients, providing valuable insights for diagnosis and treatment monitoring. This complementary method to traditional targeted biochemical assays offers several advantages, including the capacity to detect a wide range of metabolites from a single specimen and the potential for retrospective data analysis. The integration of LC-HRMS into routine biochemical genetics laboratories can enhance diagnostic yield and deepen our understanding of rare metabolic disorders, ultimately leading to improved patient outcomes.

## Research funding

The project was funded by the Endowment fund of the Department of Laboratory Medicine, Boston Children's Hospital.

## CRediT authorship contribution statement

**Rafael Garrett:** Writing – review & editing, Writing – original draft, Methodology, Investigation, Formal analysis, Data curation, Conceptualization. **Sara Pickett:** Methodology, Formal analysis, Data curation. **Melinda J. Peters:** Writing – review & editing, Investigation. **Khadija Belhassan:** Writing – review & editing, Investigation. **Adam S. Ptolemy:** Writing – review & editing, Investigation. **Roy W.A. Peake:** Writing – review & editing, Writing – original draft, Supervision, Funding acquisition, Conceptualization.

## Declaration of competing interest

None.

## Data Availability

Data will be made available on request.

## References

[1] Ferreira C.R., van Karnebeek C.D.M., Vockley J., Blau N. (2019). A proposed nosology of inborn errors of metabolism. Genet. Med..

[2] https://www.hrsa.gov/advisory-committees/heritable-disorders/rusp.

[3] Liu N., Xiao J., Gijavanekar C., Pappan K.L., Glinton K.E., Shayota B.J., Kennedy A.D., Sun Q., Sutton V.R., Elsea S.H. (2021). Comparison of untargeted Metabolomic profiling vs traditional metabolic screening to identify inborn errors of metabolism. JAMA Netw. Open.

[4] Zurynski Y., Deverell M., Dalkeith T., Johnson S., Christodoulou J., Leonard H., Elliott E.J. (2017). APSU rare diseases impacts on families study group. Australian children living with rare diseases: experiences of diagnosis and perceived consequences of diagnostic delays. Orphanet J Rare Dis..

[5] Miller M.J., Kennedy A.D., Eckhart A.D., Burrage L.C., Wulff J.E., Miller L.A., Milburn M.V., Ryals J.A., Beaudet A.L., Sun Q., Sutton V.R., Elsea S.H. (2015). Untargeted metabolomic analysis for the clinical screening of inborn errors of metabolism. J. Inherit. Metab. Dis..

[6] Kennedy A.D., Miller M.J., Beebe K., Wulff J.E., Evans A.M., Miller L.A., Sutton V.R., Sun Q., Elsea S.H. (2016). Metabolomic profiling of human urine as a screen for multiple inborn errors of metabolism. Genet. Test. Mol. Biomarkers.

[7] Coene K.L.M., Kluijtmans L.A.J., van der Heeft E., Engelke U.F.H., de Boer S., Hoegen B., Kwast H.J.T., van de Vorst M., Huigen M.C.D.G., Keularts I.M.L.W., Schreuder M.F., van Karnebeek C.D.M., Wortmann S.B., de Vries M.C., Janssen M.C.H., Gilissen C., Engel J., Wevers R.A. (2018). Next-generation metabolic screening: targeted and untargeted metabolomics for the diagnosis of inborn errors of metabolism in individual patients. J. Inherit. Metab. Dis..

[8] Haijes H.A., Willemsen M., Van der Ham M., Gerrits J., Pras-Raves M.L., Prinsen H.C.M.T., Van Hasselt P.M., der Velden MGM De Sain-van, Verhoeven-Duif N.M., JJM Jans (2019). Direct infusion based metabolomics identifies metabolic disease in patients’ dried blood spots and plasma. Metabolites.

[9] Wevers R.A., Blau N. (2018). Think big - think omics. J. Inherit. Metab. Dis..

[10] Willems A.P., van der Ham M., Schiebergen-Bronkhorst B.G.M., van Aalderen M., de Barse M.M.J., De Gruyter F.E., van Hoek I.N., Pras-Raves M.L., MGM de Sain van der Velden, HCMT Prinsen, Verhoeven-Duif N.M., JJM Jans (2023). A one-year pilot study comparing direct-infusion high resolution mass spectrometry based untargeted metabolomics to targeted diagnostic screening for inherited metabolic diseases. Front. Mol. Biosci..

[11] Strauss K.A., Puffenberger E.G., Carson V.J., Adam M.P., Feldman J., Mirzaa G.M. (2006 Jan 30). GeneReviews® [Internet].

[12] Strauss K.A., Morton D.H. (2003). Branched-chain ketoacyl dehydrogenase deficiency: maple syrup disease. Curr. Treat. Options. Neurol..

[13] Oglesbee D., Sanders K.A., Lacey J.M., Magera M.J., Casetta B., Strauss K.A., Tortorelli S., Rinaldo P., Matern D. (2008). Second-tier test for quantification of alloisoleucine and branched-chain amino acids in dried blood spots to improve newborn screening for maple syrup urine disease (MSUD). Clin. Chem..

[14] Puliyanda D.P., Harmon W.E., Peterschmitt M.J., Irons M., Somers M.J. (2004). Utility of hemodialysis in maple syrup urine disease. Pediatr. Nephrol..

[15] Strauss K.A., Carson V.J., Soltys K., Young M.E., Bowser L.E., Puffenberger E.G., Brigatti K.W., Williams K.B., Robinson D.L., Hendrickson C., Beiler K., Taylor C.M., Haas-Givler B., Chopko S., Hailey J., Muelly E.R., Shellmer D.A., Radcliff Z., Rodrigues A., Loeven K., Heaps A.D., Mazariegos G.V., Morton D.H. (2020). Branched-chain α-ketoacid dehydrogenase deficiency (maple syrup urine disease): treatment, biomarkers, and outcomes. Mol. Genet. Metab..

[16] Schadewaldt P., Bodner-Leidecker A., Hammen H.W., Wendel U. (1999). Significance of L-alloisoleucine in plasma for diagnosis of maple syrup urine disease. Clin. Chem..

[17] Chen S., Hoene M., Li J., Li Y., Zhao X., Häring H.U., Schleicher E.D., Weigert C., Xu G., Lehmann R. (2013). Simultaneous extraction of metabolome and lipidome with methyl tert-butyl ether from a single small tissue sample for ultra-high performance liquid chromatography/mass spectrometry. J. Chromatogr. A.

[18] Garrett R., Ptolemy A.S., Pickett S., Kellogg M.D., Peake R.W.A. (2024). Untargeted metabolomics for inborn errors of metabolism: development and evaluation of a sustainable reference material for correcting inter-batch variability. Clin. Chem..

[19] Peake R.W.A., Law T., Hoover P.N., Gaewsky L., Shkreta A., Kellogg M.D. (2013). Improved separation and analysis of plasma amino acids by modification of the MassTrakTM AAA solution Ultraperformance® liquid chromatography method. Clin. Chim. Acta.

[20] Adams K.J., Pratt B., Bose N., Dubois L.G., St John-Williams L., Perrott K.M., Ky K., Kapahi P., Sharma V., MJ MacCoss, Moseley M.A., Colton C.A., BX MacLean, Schilling B., Thompson J.W., Alzheimer’s Disease Metabolomics Consortium (2020). Skyline for small molecules: a unifying software package for quantitative metabolomics. J. Proteome Res..

[21] Piskláková Barbora, Friedecká Jaroslava, Ivanovová Eliška, Hlídková Eva, Bekárek Vojtěch, Prídavok Matúš, Kvasnička Aleš, Adam Tomáš, Friedecký David (2023). Rapid and efficient LC-MS/MS diagnosis of inherited metabolic disorders: a semi-automated workflow for analysis of organic acids, acylglycines, and acylcarnitines in urine. Clin. Chem. Lab. Med..

[22] Broadhurst D.I. (2024). Exploration & Data Cleaning (version 1) Zendono.

[23] Demsar J., Curk T., Erjavec A., Gorup C., Hocevar T., Milutinovic M., Mozina M., Polajnar M., Toplak M., Staric A., Stajdohar M., Umek L., Zagar L., Zbontar J., Zitnik M., Zupan B. (2013). Orange: data mining toolbox in Python. J. Mach. Learn. Res..

[24] Blackburn P.R., Gass J.M., Vairo F.P.E., Farnham K.M., Atwal H.K., Macklin S., Klee E.W., Atwal P.S. (2017). Maple syrup urine disease: mechanisms and management. Appl. Clin. Genet..

[25] Fisher C.W., Fisher C.R., Chuang J.L., Lau K.S., Chuang D.T., Cox R.P. (1993). Occurrence of a 2-bp (AT) deletion allele and a nonsense (G-to-T) mutant allele at the E2 (DBT) locus of six patients with maple syrup urine disease: multiple-exon skipping as a secondary effect of the mutations. Am. J. Hum. Genet..

[26] Khalifa O.A., Imtiaz F., Ramzan K., Zaki O., Gamal R., Elbaik L., Rihan S., Salam E., Abdul-Mawgoud R., Hassan M., Hassan N., Saleh E., Seoudi D., Moustafa A.S. (2020). Genotype-phenotype correlation of 33 patients with maple syrup urine disease. Am. J. Med. Genet. A.

[27] Pode-Shakked N., Korman S.H., Pode-Shakked B., Landau Y., Kneller K., Abraham S., Shaag A., Ulanovsky I., Daas S., Saraf-Levy T., Reznik-Wolf H., Vivante A., Pras E., Almashanu S., Anikster Y. (2020). Clues and challenges in the diagnosis of intermittent maple syrup urine disease. Eur. J. Med. Genet..

[28] Atwal P.S., Macmurdo C., Grimm P.C. (2015). Haemodialysis is an effective treatment in acute metabolic decompensation of maple syrup urine disease. Mol. Genet. Metab. Rep..

[29] Torres C.L., Sardela V.F., Scalco F.B., de Neto F.R.A., Garrett R. (2022). Development and application of a test mixture for untargeted liquid chromatography-mass spectrometry analysis of urine samples. Quim Nova.

[30] Szuch E., Auriemma J. (2018 Jul 10). Recurrent encephalopathy during febrile illnesses in a 6-year-old boy. Glob. Pediatr. Health.

[31] Chuang D.T., Chuang J.L., Wynn R.M. (2006). Lessons from genetic disorders of branched-chain amino acid metabolism. J. Nutr..

